# Doxorubicin Embedded into Nanofibrillated Bacterial Cellulose (NFBC) Produces a Promising Therapeutic Outcome for Peritoneally Metastatic Gastric Cancer in Mice Models via Intraperitoneal Direct Injection

**DOI:** 10.3390/nano11071697

**Published:** 2021-06-28

**Authors:** Hidenori Ando, Takashi Mochizuki, Amr S. Abu Lila, Shunsuke Akagi, Kenji Tajima, Kenji Fujita, Taro Shimizu, Yu Ishima, Tokuo Matsushima, Takatomo Kusano, Tatsuhiro Ishida

**Affiliations:** 1 Department of Pharmacokinetics and Biopharmaceutics, Institute of Biomedical Sciences, Tokushima University, Tokushima 770-8505, Japan; h.ando@tokushima-u.ac.jp (H.A.); c401503025@tokushima-u.ac.jp (T.M.); c401603010@tokushima-u.ac.jp (S.A.); kenji-fujita@taiho.co.jp (K.F.); shimizu.tarou@tokushima-u.ac.jp (T.S.); ishima.yuu@tokushima-u.ac.jp (Y.I.); 2 Department of Pharmaceutics and Industrial Pharmacy, Faculty of Pharmacy, Zagazig University, Zagazig 44519, Egypt; amr_selim78@yahoo.com; 3 Department of Pharmaceutics, College of Pharmacy, Hail University, Hail 81442, Saudi Arabia; 4 Faculty of Engineering, Hokkaido University, Hokkaido 060-8628, Japan; ktajima@eng.hokudai.ac.jp; 5 Kusano Sakko Inc., Hokkaido 067-0063, Japan; t-matsushima@kusanosk.co.jp (T.M.); tk@kusanosk.co.jp (T.K.)

**Keywords:** nanofibrillated bacterial cellulose, bacterial cellulose, peritoneally disseminated gastric cancer, doxorubicin, intraperitoneal chemotherapy

## Abstract

Natural materials such as bacterial cellulose are gaining interest for their use as drug-delivery vehicles. Herein, the utility of nanofibrillated bacterial cellulose (NFBC), which is produced by culturing a cellulose-producing bacterium (*Gluconacetobacter intermedius* NEDO-01) in a medium supplemented with carboxymethylcellulose (CMC) that is referred to as CM-NFBC, is described. Recently, we demonstrated that intraperitoneal administration of paclitaxel (PTX)-containing CM-NFBC efficiently suppressed tumor growth in a peritoneally disseminated cancer xenograft model. In this study, to confirm the applicability of NFBC in cancer therapy, a chemotherapeutic agent, doxorubicin (DXR), embedded into CM-NFBC, was examined for its efficiency to treat a peritoneally disseminated gastric cancer via intraperitoneal administration. DXR was efficiently embedded into CM-NFBC (DXR/CM-NFBC). In an in vitro release experiment, 79.5% of DXR was released linearly into the peritoneal wash fluid over a period of 24 h. In the peritoneally disseminated gastric cancer xenograft model, intraperitoneal administration of DXR/CM-NFBC induced superior tumor growth inhibition (TGI = 85.5%) by day 35 post-tumor inoculation, compared to free DXR (TGI = 62.4%). In addition, compared with free DXR, the severe side effects that cause body weight loss were lessened via treatment with DXR/CM-NFBC. These results support the feasibility of CM-NFBC as a drug-delivery vehicle for various anticancer agents. This approach may lead to improved therapeutic outcomes for the treatment of intraperitoneally disseminated cancers.

## 1. Introduction

The advent of nanotechnology has caused a profound impact on cancer therapeutics and diagnostics. Recently, there has been considerable progress in the development of nano-based drug delivery system that can specifically target the tumor site while minimizing damage to normal cells. Furthermore, a great shift in paradigm in regard to the use of nanobiomaterials over conventional systems has been observed. Currently, several types of biomaterials such as proteins, lipids, polysaccharides, polymers, etc., are widely used nowadays to deliver drugs to the tumor site [1].

Cellulose is a naturally produced homopolysaccharide composed of *β*-D-glucopyranose units linked by *β*-1,4-glycosidic bonds, and is mainly synthesized by plants [2,3]. Additionally, cellulose is also synthesized by strains of bacteria such as *Agrobacterium*, *Alcaligenes*, *Pseudomonas*, *Rhizobium*, *Sarcina*, and *Gluconacetobacter* [4,5,6,7]. Bacterial cellulose (BC), produced by such aerobic bacteria under static culture conditions, has recently received increased attention owing to unique physiochemical properties that are not found in plant cellulose. BC is more chemically pure and contains neither hemicellulose nor lignin. In addition, BC has nanometric characteristics, higher levels of water retention, higher levels of biocompatibility, and greater tensile strength that is the result of a greater amount of polymerization and an ultrafine network architecture [8,9,10].

By virtue of their unique properties, cellulose nanofibers (CNFs) such as BC have recently attracted a considerable amount of attention as drug delivery vehicles [11,12,13]. CNF aerogels, with favorable floatability and mucoadhesive properties, prepared by freeze-drying methods, were efficiently used for formulating gastroretentive drug delivery systems with improved bioavailability [11]. The potential of CNFs as delivery carriers for poorly soluble drugs has also been described [13]. Nevertheless, most studies have focused mainly on exploiting the unique physicochemical properties of CNFs that enhance drug loading in vitro, but little consideration has been given to the in vivo therapeutic efficacy of formulated drug-loaded CNFs.

In our recent study, paclitaxel (PTX)-embedded nanofibrillated bacterial cellulose (NFBC) were prepared for intraperitoneal administration and studied for their therapeutic efficacy on peritoneally disseminated gastric cancer in a xenograft nude mouse model. The results were compared with those from treatment with Taxol^®^ (TAX) and Abraxane^®^, which are generally used in clinical settings. Systemic side effects such as those induced by the use of TAX were not present [14]. This represented the first report of a therapeutic effect of a BC-based anticancer drug formulation in a small animal model. The NFBC was synthesized using waste glycerol from a novel strain of *Gluconacetobacter intermedius* (named NEDO-01) isolated from the surfaces of fruits [15]. The NFBC showed potential for use as a drug-carrier vehicle. Under optimum *Gluconacetobacter* culture conditions, NEDO-01 produces a large amount of NFBC [15], which significantly reduces manufacturing cost and increases the possibility for practical application. By synthesizing NFBC in a medium supplemented with either carboxymethylcellulose (CMC) or hydroxypropylcellulose (HPC), the production of either hydrophilic NFBC (CM-NFBC) or amphiphilic NFBC (HP-NFBC) is possible [16]. In addition, in contrast to conventional top-down types of CNFs, which are obtained via mechanical treatment of wood pulp that contains submicro- or micro-sized fibers [17], NFBC synthesized via a bottom-up process from low-molecular-weight biomass via the use of NEDO-01 contains only trace amounts of submicro- or micro-sized fibers. Furthermore, manipulation of the culture conditions for the preparation of homogeneous nanofibers may be used to optimize the production of NFBC [15,16].

For this study, to extend the usability of NFBC as a drug-delivery vehicle, doxorubicin (DXR) was embedded in CM-NFBC (DXR/CM-NFBC). The in vivo therapeutic effects of the embedded material in a peritoneally disseminated gastric cancer xenograft mouse model were assessed.

## 2. Materials and Methods

### 2.1. Materials

DXR (Adryacin^®^) was purchased from Kyowa Kirin (Tokyo, Japan). CMC was kindly provided by Daicel (Osaka, Japan). *D*-luciferin potassium salt and bovine serum albumin (BSA) were purchased from FUJIFILM Wako Pure Chemical Corporation (Osaka, Japan). All other reagents were of analytical grade.

### 2.2. Animals

BALB/c mice (5-week-old, male) and BALB/c *nu*/*nu* mice (5-week-old, male) weighing 20–22 g were purchased from Japan SLC (Shizuoka, Japan). The small animals had free access to water and mouse chow and were housed under controlled environmental conditions (constant temperature, humidity and a 12 h dark/light cycle). All animal experiments were evaluated and approved by the Animal and Ethics Review Committee of Tokushima University (the approval code: T2019-47).

### 2.3. Preparation of CM-NFBC

CM-NFBC was prepared from an identified strain, NEDO-01, as previously described [16]. Briefly, the bacteria were inoculated into 10 mL Hestrin and Schramm’s (HS) medium and incubated at 30 °C for 3 days under static conditions. The pellicle (3D network structure) formed at the air/liquid interface was pressed using a sterilized toothpick to isolate the bacteria. The culture medium containing bacteria (1 mL) was then inoculated into 10 mL of HS medium and incubated at 30 °C for 3 days under static conditions. The cultured medium containing bacteria (5 mL) was further inoculated into 100 mL HS medium supplemented with 2% *w*/*v* CMC and incubated at 30 °C on a rotating shaker (BR-43FL, TAITEC, Saitama, Japan) at 150 rpm. The cellulose products were collected by centrifugation (CR20GIII, Hitachi Koki, Tokyo, Japan) and dispersed into 1% *w*/*v* aqueous NaOH followed by incubation for 2 h at 70 °C with shaking, to lyse the remaining bacterial cells. After strong alkaline treatment, CM-NFBC was collected by centrifugation and washed with deionized water until it reached a pH of 6–7.

### 2.4. Preparation of DXR/CM-NFBC

DXR/CM-NFBC was prepared by mixing the DXR solution (2 mg/mL in Saline) with an equal volume of CM-NFBC (0.1, 0.2, 0.4, or 0.8% *w*/*v*). The concentration of DXR in the resultant DXR/CM-NFBC was adjusted to 1 mg/mL, and that of CM-NFBC was adjusted to 0.05, 0.1, 0.2, or 0.4% *w*/*v*.

### 2.5. Determining the Drug-Embedded Ratio in CM-NFBC

The DXR/CM-NFBC was centrifuged at 4 °C for 3 min at 15,000× *g* using a micro-centrifuge system (KUBOTA, Osaka, Japan) to determine the retained amount of DXR. The supernatant was collected and diluted with MeOH (10 µL of supernatant with 100 µL of MeOH). The fluorescence intensity of the DXR in the supernatant samples was measured at an excitation wavelength of 470 nm and an emission wavelength of 590 nm using EnSpire™ (PerkinElmer, Waltham, MA, USA). The DXR concentration in the supernatant was determined from a preconstructed standard curve of DXR at various concentrations. The embedded ratio (%) of the DXR in the prepared DXR/CM-NFBC was then calculated using the following formula:

$$\mathrm{DXR}\;embedded\;ratio\;\left(\%\right)=\left(\frac{{\mathrm{DXR}}_{initial}-{\mathrm{DXR}}_{supernatant}}{{\mathrm{DXR}}_{initial}}\;X\;100\right)$$

DXR*_initial_*: a total initial amount of DXR

DXR*_supernatant_*: an amount of free DXR in the supernatant

### 2.6. Release of DXR from DXR/CM-NFBC In Vitro

In vitro drug release from DXR/CM-NFBC formulations was evaluated in peritoneal wash fluid. In order to obtain peritoneal wash fluid, BALB/c mice were euthanized under deep anesthesia followed by intraperitoneal injection with 5 mL of saline to wash the peritoneal cavity, and then the peritoneal wash fluid was collected.

For in vitro release experiments, 500 µL of DXR/CM-NFBC was centrifuged at 4 °C for 3 min at 15,000× *g*, and the supernatant was discarded. Peritoneal wash fluid was then added onto the DXR/CM-NFBC pellet dropwise to a total volume of 1 mL. At selected time points post-incubation at 37 °C (0.5, 1, 2, 6 and 24 h), 100 µL of the supernatant was collected and thoroughly mixed with 300 µL of acetonitrile. After centrifugation (8000× *g*, 4 °C, 15 min), the supernatant (200 µL) was collected and mixed with 200 µL of water. DXR content was analyzed using a HPLC system (Shimadzu, Kyoto, Japan). The analysis was accomplished using a 4.6 × 150 mm C18 column (TSKgel ODS-100V) that was maintained at 35 °C with a mobile phase that consisted of acetonitrile and water (32:68, *v*/*v*; pH = 2.6). The flow rate was maintained at 1 mL/min, and the column effluent was monitored with an ultraviolet detector at 254 nm. The injection volume was 10 µL, and the retention time for DXR was 6.8 min. The DXR concentration was determined from a preconstructed calibration curve of DXR at various concentrations.

### 2.7. Preparation of Peritoneally Disseminated Gastric Cancer Xenograft Mouse Model

NCI-N87 human gastric carcinoma expressing firefly luciferase (NCI-N87-Luc, 128443) was purchased from Summit Pharmaceuticals International (Tokyo, Japan). The cells were cultured in RPMI-1640 medium (FUJIFILM Wako Pure Chemical Corporation) supplemented with 10% of heat-inactivated fetal bovine serum (COSMO BIO, Tokyo, Japan), 100 units/mL penicillin and 100 µg/mL streptomycin (ICN Biomedicals, Costa Mesa, CA, USA) in a 5% CO_2_/air incubator at 37 °C.

To prepare a peritoneally metastatic gastric cancer mouse model, NCI-N87-Luc cells were intraperitoneally inoculated into BALB/c *nu*/*nu* mice (5 × 10^6^ cells/mouse). Development and reduction of NCI-N87-Luc cells in the peritoneal cavity was confirmed using an in vivo imaging system (IVIS, Xenogen, Alameda, CA, USA) following an injection of *D*-luciferin potassium salt (100 µL of 7.5 mg/mL) under aesthesis with isoflurane inhalation. At 5 min post injection, bioluminescence derived from NCI-N87-Luc cells was observed with a CCD camera (30 s for the exposure time). The region of interest (ROI) in the bioluminescence was calculated and shown as bioluminescence intensity (BLI).

### 2.8. Evaluating the Tumor-Growth Inhibitory Effect of DXR/CM-NFBC on a Peritoneally Disseminated NCI-N87-Luc Xenograft Mouse Model

Peritoneally disseminated NCI-N87-Luc model mice were intraperitoneally injected with 4 doses of either free DXR or a formulation of DXR/CM-NFBC (0.5 mg DXR/kg/day) once a week from Day 8 post-tumor inoculation. At selected post-tumor inoculation time-points (Days 7, 14, 21, 28, and 35), luciferase activity of the inoculated tumor cells in the peritoneal cavity was monitored with IVIS as aforementioned, and the body weights of the treated mice were recorded twice weekly. The antitumor effect was assessed in terms of both the bioluminescence intensity (BLI) and the percentage of tumor growth inhibition [TGI (%)]. The TGI was calculated using the following formula:

TGI %=1−BLItreatedBLIcontrol ×100

BLI*_treated_*: A BLI in mouse treated with free DXR or DXR/CM-NFBC

BLI*_control_*: A BLI in mouse non-treated

As a control, the model mice were intraperitoneally injected with 4 doses of CM-NFBC alone once a week from Day 8 post-tumor inoculation in amounts equal to the injections of DXR/CM-NFBC. At selected time points (Days 7, 13, 19, 25, and 31), luciferase activity of the peritoneally inoculated tumors was observed, and the BLI was calculated. At selected time points (Days 8, 14, 20, and 26), body weights of the mice were recorded.

### 2.9. Statistical Analysis

Statistical differences between the groups were evaluated by analysis of variance (ANOVA) with the Tukey post-hoc test, to explore the mean differences between pairs of groups, using Prism 8 software (GraphPad Software, San Diego, CA, USA). All values are reported as the mean ± S.D. The levels of significance were set at * *p* < 0.05, ** *p* < 0.01, and *** *p* < 0.001.

## 3. Results

### 3.1. Preparation of DXR/CM-NFBC Formulation

**Figure 1:**
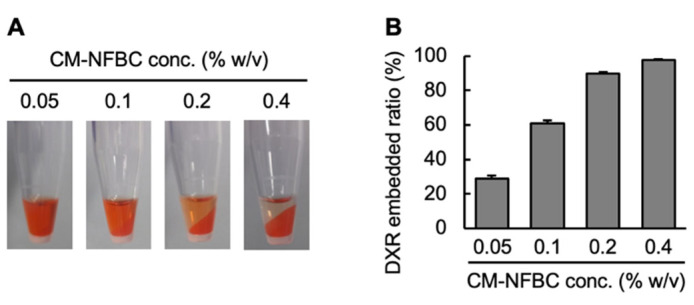
Preparation of DXR-embedding into CM-NFBC (DXR/CM-NFBC). DXR/CM-NFBC was prepared by mixing a DXR solution (2 mg/mL) with an equal volume of different concentrations of CM-NFBC (0.1, 0.2, 0.4, or 0.8% *w*/*v*). The concentration of DXR in the resultant DXR/CM-NFBC was adjusted to 1 mg/mL, and that of CM-NFBC was adjusted to 0.05, 0.1, 0.2, or 0.4% *w*/*v*. (**A**) DXR/CM-NFBC prepared with different concentrations of CM-NFBC. Photos were taken after centrifugation (15,000× *g*, 4 °C, 3 min). (**B**) Percentage of DXR embedded in the CM-NFBC. Data represent the mean ± SD (*n* = 6).

DXR is a widely used chemotherapeutic agent that was employed as an amphiphilic model drug for the preparation of a chemotherapeutic agent that could be embedded into CM-NFBC. The first step was to optimize the DXR/CM-NFBC formulation. To ensure efficient embedding, DXR was mixed with different concentrations of CM-NFBC (0.05, 0.1, 0.2, or 0.4% *w*/*v*). Figure 1A depicts the images of the prepared DXR/CM-NFBC after centrifugation. CM-NFBC was precipitated by centrifugation. At the lower CM-NFBC concentrations (0.05 or 0.1% *w*/*v*), strong red colors were observed in the supernatant, which indicated the presence of free/unembedded DXR. At relatively higher CM-NFBC concentrations (0.2 or 0.4% *w*/*v*), however, either few or no red colors were observed in the supernatant, which indicated that almost 100% of the added DXR had been embedded in the CM-NFBC. The DXR embedded ratios within each aliquot of DXR/CM-NFBC were calculated by measuring the fluorescent intensity of free DXR in the supernatant (Figure 1B). The DXR embedded ratios were directly proportional to CM-NFBC concentrations; the ratio was increased to 97.8% at 0.4% *w*/*v* CM-NFBC. The optimal mixture of DXR (2 mg/mL) and CM-NFBC (0.4% *w*/*v*) was adopted for further experiments.

### 3.2. Release of Embedded DXR from the DXR/CM-NFBC Formulation in Peritoneal Fluid

**Figure 2:**
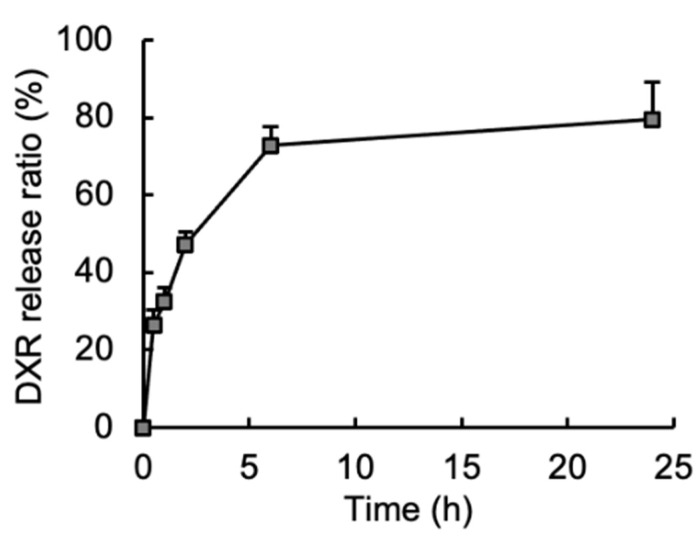
In vitro DXR release from DXR/CM-NFBC in peritoneal wash fluid. DXR/CM-NFBC (500 µL) was centrifuged and the supernatant was discarded. Peritoneal wash fluid was then added dropwise onto the DXR/CM-NFBC precipitates to a total volume of 1 mL. At selected time points post-incubation at 37 °C (0.5, 1, 2, 6 and 24 h), 100 µL of the supernatant was collected, and the DXR content was analyzed via HPLC. Data represent the mean ± SD (*n* = 6).

One of the main purposes of this study was to apply the DXR/CM-NFBC formulation into the peritoneal cavity to treat peritoneally disseminated gastric cancer by intraperitoneal injection. Accordingly, the drug-release properties of DXR/CM-NFBC were evaluated via the incubation of DXR/CM-NFBC in the peritoneal wash fluid obtained from normal mice (Figure 2). Following 0.5 h of incubation, 26.6% of DXR was rapidly released from the formulation. Then, the release ratio reached a maximum level (79.5%) after 24 h of incubation. DXR/CM-NFBC appeared to achieve a sustained release of embedded DXR following intraperitoneal administration.

### 3.3. Growth Inhibitory Effect of an Intraperitoneally Injected DXR/CM-NFBC Formulation on Peritoneally Inoculated NCI-N87-Luc Tumors

**Figure 3:**
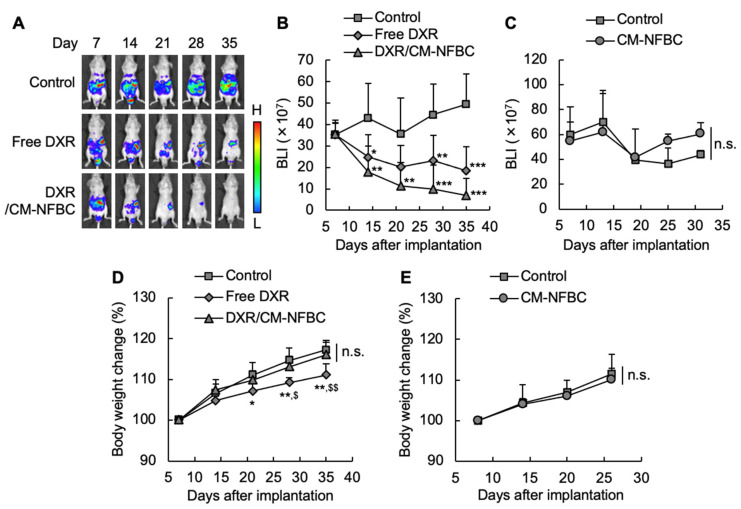
Growth inhibitory effect of an intraperitoneally injected DXR/CM-NFBC formulation on peritoneally inoculated NCI-N87-Luc tumors. Peritoneally disseminated gastric cancer (NCI-N87-Luc) xenograft model mice were intraperitoneally injected with four doses of either free DXR, the DXR/CM-NFBC formulation (0.5 mg DXR/kg/day), or CM-NFBC alone once a week from Day 8 post-tumor-inoculation. At selected time points post-tumor-inoculation, luciferase activity of the peritoneally metastatic tumors was monitored with IVIS. (**A**) Bioluminescence intensity (BLI) of peritoneally inoculated NCI-N87-Luc cells in each mouse treated with various formulations. Photos are typical examples from six mice. (**B**) The BLI of interest in the mice treated with various formulations. (**C**) The BLI of interest in the mice treated with CM-NFBC alone. (**D**) Body weight changes in the mice treated with various formulations. (**E**) Body weight changes in the mice treated with CM-NFBC alone. Data represent the mean ± SD (*n* = 6, * *p* < 0.05, ** *p* < 0.01, *** *p* < 0.001 vs. control, ^$^ *p* < 0.05, ^$$^ *p* < 0.01 vs. DXR/CM-NFBC, n.s.: no significance).

To verify the tumor growth inhibitory effects, DXR/CM-NFBC was intraperitoneally injected into the mice in which NCI-N87-Luc cells had previously been peritoneally inoculated. The growth of inoculated cells was manifested by the increase in luciferase activity derived from NCI-N87-Luc cells before the injection of DXR/CM-NFBC. In the control group, luciferase activity gradually increased with time (Figure 3A,B), which indicated a steady growth of peritoneally inoculated cells. In both the free DXR and DXR/CM-NFBC groups (Figure 3A,B), luciferase activity was significantly decreased with time, suggesting that these treatments suppressed the number of inoculated cells in the peritoneal cavity. DXR/CM-NFBC treatment tended to produce a higher suppression effect than free DXR treatment. Treatment with drug-free CM-NFBC induced no tumor growth suppression (Figure 3C). To gain further insight, the TGI (%) was calculated from the values of BLI shown in Figure 3B (Table 1). These calculations confirmed that the treatment with either free DXR or DXR/CM-NFBC significantly decreased the BLI compared with that of the control group. In addition, treatment with DXR/CM-NFBC tended to induce a higher tumor growth inhibitory effect compared with treatment with free DXR. To confirm the systemic side effects of DXR/CM-NFBC, we monitored the body weight changes in peritoneally disseminated NCI-N87-Luc model mice following intraperitoneal treatment with DXR/CM-NFBC (Figure 3D). Treatment with free DXR clearly hampered efforts to increase the body weight of the treated mice, which indicated that the treatment with free DXR had induced the systemic side effects even with intraperitoneal treatment. In contrast to the control group, intraperitoneal treatment with DXR/CM-NFBC induced no significant body weight loss, and no body weight loss was observed in mice treated with drug-free CM-NFBC (Figure 3E).

## 4. Discussion

**Figure 4:**
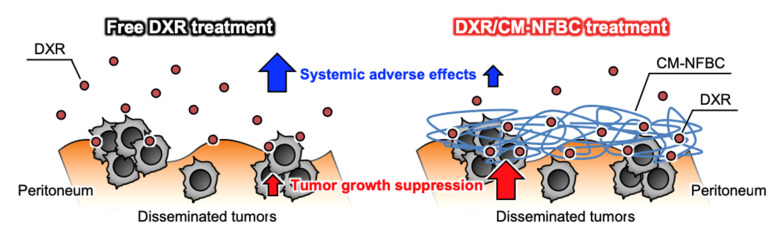
Schematic diagram of free DXR and DXR/CM-NFBC after intraperitoneal injection into a peritoneally disseminated tumor-xenograft mouse model.

To date, BC has gained an ever-increasing amount of attention in the fields of biomedical and tissue engineering, which is a testament to qualities such as biocompatibility, flexibility, and mechanical strength [13,18,19]. However, only a few reports have mentioned the utility of BC as a drug-delivery vehicle. We recently reported that CM-NFBC, produced by culturing the strain identified as NEDO-01 under optimized conditions [15,16], could efficiently entrap the cytotoxic agent PTX, and the intraperitoneal administration of PTX-embedded CM-NFBC has shown superior therapeutic effects on a peritoneally disseminated gastric cancer xenograft mouse model [14]. To expand our work, in the present study we selected another anticancer agent, DXR, which is widely used to treat ovarian, bladder, breast, lung, thyroid, and stomach cancers [20,21,22]. CM-NFBC efficiently embedded DXR (Figure 1), and intraperitoneal administration of the formulation tended to suppress the peritoneal tumor growth and body weight loss, compared with that of the free DXR formulation, in a peritoneally disseminated gastric cancer xenograft mouse model (Figure 3 and Figure 4). To the best of our knowledge, this is the first report to demonstrate the utility of CM-NFBC, not only for the delivery of a hydrophobic chemotherapeutic agent such as PTX, but also as a vehicle for an amphiphilic chemotherapeutic agent such as DXR.

Adequate drug release from a delivery vehicle at the target site of action is the key to gaining maximum therapeutic effect and to minimizing the side effects of embedded/encapsulated drugs [23]. DXR/CM-NFBC had efficiently released 60% of the embedded DXR into the peritoneal wash fluid within 6 h (Figure 2). The wash fluid mimics the in vivo environment of the target site of peritoneally disseminated gastric cancer xenograft mouse models. Therefore, the rapid and sustained release of DXR from the formulation in the present in vitro release study of a peritoneally disseminated cancer model (Figure 2) is a major indicator for the promise of a therapeutic effect with no body weight change (Figure 3).

The three-dimensional network structure of hydrogels such as CNFs tends to incorporate drugs and promote sustained release, which potentiates the antitumor effects and limits the undesirable side effects [24]. CM-NFBC is composed of hydrophilic cellulose networks with semi-micro sizes of pores and is characterized by relatively uniform nanofibers with a diameter of ca. 20 nm [15]. Due to such a unique structure and characteristics, DXR was efficiently trapped by the hydrophilic cellulose network when vigorously mixed with CM-NFBC. Furthermore, the -COOH groups in the hydrogel were ionized into carboxyl ions (-COO^−^) in a neutral condition that expanded the network structure via electrostatic repulsion, which allowed the absorption of water into the hydrogel [25,26]. CM-NFBC is composed of nanofibrillated cellulose, and when modified with CMC on the surfaces of the nanofibers, this formulation contains -COOH groups owing to the high frequency of mixing [15,16]. Following the intraperitoneal injection of DXR/CM-NFBC, these -COOH groups would likely expand the cellulose network of NFBC in the peritoneal cavity and absorb water, which should enhance the release of DXR from the DXR/CM-NFBC formulation.

Anthracycline anticancer drugs, such as DXR, daunorubicin, and epirubicin, are an important class of chemotherapeutic agents for the treatment of a wide variety of solid tumors. Notably, DXR is a key chemotherapeutic drug for cancer treatment, although its clinical use is limited by acute and chronic adverse events. In a retrospective analysis of more than 4000 patients receiving DXR, 2.2% developed clinical signs and symptoms of congestive heart failure [27]. The toxicity that DXR exhibits in cardiomyocytes is related to free radical formation caused by the metabolism of DXR after accumulation in the heart [28], which means that a transfer of DXR into the circulation and its subsequent accumulation in the heart would likely induce severe cardiotoxicity. Indeed, the intraperitoneal injection of free DXR caused substantial body weight loss in a mouse model (Figure 3D) probably due to the quick distribution of DXR into circulation. By contrast, an intraperitoneal injection of DXR/CM-NFBC did not decrease the body weight of the mice compared with the non-treatment control mice (Figure 3D), which suggests a relatively lower concentration of DXR in the peritoneal cavity and blood circulation due to the sustained release of DXR from the DXR/CM-NFBC formulation (Figure 2).

In our previous study, when PTX, a hydrophobic anticancer drug, was embedded in CM-NFBC it was barely released from the formulation in the peritoneal wash fluid; less than 3% of embedded PTX was released even after 24 h of incubation [14]. Taken together with the results shown in Figure 1 and Figure 2, it appears that the physicochemical properties of drugs mainly govern their levels of embedding into CM-NFBC as well as their release from the formulation. Both amphiphilic drugs such as DXR and hydrophobic drugs such as PTX are strongly embedded into CM-NFBC. Amphiphilic drugs such as DXR might be quickly released from the formulation, while hydrophobic drugs such as PTX might be retained within the formulation.

The in vivo imaging study showed that DXR/CM-NFBC successfully tended to increase the suppressive effect of DXR on tumor growth in a peritoneally disseminated gastric cancer xenograft mouse model compared with the free DXR formulation (Figure 3A,B). Intraperitoneal chemotherapy has recently been introduced as a smart strategy to treat peritoneally disseminated cancers, because intraperitoneal chemotherapy can directly expose metastatic tumors to the drugs of interest [29,30]. Nevertheless, intraperitoneally injected anticancer drugs, notably hydrophilic/amphiphilic ones such as DXR and cisplatin, tend to be rapidly cleared from the peritoneal cavity via the circulatory system [31], and thus no effective treatment with intraperitoneal chemotherapy has yet improved the survival rates of patients with peritoneal dissemination of cancers. Our drug/CM-NFBC formulation could represent a suitable pharmaceutical formulation for clinical settings in achieving the long peritoneal retention of hydrophilic/amphiphilic agents following intraperitoneal injection.

## 5. Conclusions

A novel formulation, DXR/CM-NFBC, was successfully prepared by embedding the amphiphilic anticancer drug, DXR. This formulation showed a somewhat higher therapeutic efficacy in a peritoneally metastatic gastric cancer xenograft mouse model compared with the use of free DXR. We collectively demonstrated that our NFBC is a potentially powerful drug delivery vehicle for various anticancer agents used in the treatment of peritoneally disseminated cancers via intraperitoneal injection.

## Figures and Tables

**Table 1:** Tumor growth inhibitory effects of the DXR/CM-NFBC formulation. (* *p* < 0.05, ** *p* < 0.01, *** *p* < 0.001 vs. control).

Treatment	Day 14		Day 21		Day 28		Day 35	
	BLI (×10^7^)	TGI (%)	BLI (×10^7^)	TGI (%)	BLI (×10^7^)	TGI (%)	BLI (×10^7^)	TGI (%)
Control	43.0 ± 16.1	-----	35.6 ± 16.8	-----	44.7 ± 14.2	-----	49.3 ± 14.3	-----
Free DXR	24.9 ± 10.3 *	42.1	20.3 ± 9.8	42.9	23.2 ± 11.6 **	48.0	18.6 ± 11.1 ***	62.4
DXR/CM-NFBC	18.0 ± 12.1 **	58.2	11.4 ± 11.0 **	67.9	10.0 ± 11.3 ***	77.7	7.0 ± 8.0 ***	85.8

## Data Availability

The data presented in this study are available on request from the corresponding author.
